# *The alr-groEL1* operon in *Mycobacterium tuberculosis*: an interplay of multiple regulatory elements

**DOI:** 10.1038/srep43772

**Published:** 2017-03-03

**Authors:** Aadil H. Bhat, Deepika Pathak, Alka Rao

**Affiliations:** 1 CSIR-Institute of Microbial Technology, Sector 39-A, Chandigarh-160036, India

## Abstract

Threonylcarbamoyladenosine is a universally conserved essential modification of tRNA that ensures translational fidelity in cellular milieu. TsaD, TsaB and TsaE are identified as tRNA-A_37_-threonylcarbamoyl (t^6^A)-transferase enzymes that have been reconstituted *in vitro*, in few bacteria recently. However, transcriptional organization and regulation of these genes are not known in any of these organisms. This study describes the intricate architecture of a complex multicistronic *alr-groEL1* operon, harboring essential genes, namely *tsaD, tsaB, tsaE, groES, groEL1*, and *alr* (required for cell wall synthesis), and *rimI* encoding an N-α- acetyltransferase in *Mycobacterium tuberculosis*. Using northern blotting, RT-PCR and *in vivo* fluorescence assays, genes *alr* to *groEL1* were found to constitute an ~6.3 kb heptacistronic operon with multiple internal promoters and an I-shaped intrinsic hairpin-like *cis*-regulatory element. A strong promoter P*tsaD* within the coding sequence of *rimI* gene is identified in *M. tuberculosis*, in addition. The study further proposes an amendment in the known bicistronic *groESL1* operon annotation by providing evidence that *groESL1* is co-transcribed as sub-operon of *alr-groEL1* operon. The architecture of *alr-groEL1* operon, conservation of the genetic context and a mosaic transcriptional profile displayed under various stress conditions convincingly suggest the involvement of this operon in stress adaptation in *M. tuberculosis*.

Survival of a cell depends on its ability to maintain translational fidelity and accuracy. Transfer-RNA (tRNA), along with the large number of modifications that adorn its structure, is a major player contributing towards accurate decoding of mRNA^1^. In tRNAs that decode ANN codons, adenosine at position 37 is modified with N6-threonylcarbamoyladenosine (t^6^A). The importance of this modification is emphasized by its presence in all domains of life. This modification stabilizes the tRNA anticodon stem loop structure, enhances codon-anticodon architecture and prevents mispairing between first base of codon and third base of anticodon. In the absence of t^6^A, frame shifting is found to increase especially at sequences with tandem ANN codons^2^,^3^.

While this modification was discovered four decades ago, the enzymes involved have recently been elucidated. The core set of tRNA-A_37_-t^6^A biosynthesis pathway is conserved universally and consists of two protein families, namely TsaC/Sua5 and TsaD/Kae1/Qri7. The other participating proteins of the pathway are kingdom-specific. While TsaB and TsaE are unique to bacteria (exceptions include intracellular and symbiotic bacteria), Bud32, Pcc1 and Cgi121 are specific to eukaryotes^2^. Homologs of TsaD and TsaB are essential for growth and survival in *Escherichia coli* and *Salmonella typhimurium*. Depletion of these proteins have led to pleiotropic effects like elongation and branching of cells, unusual distribution of DNA, appearance of nucleoid etc.^4^. At least in 20% of bacterial genomes, *tsaB* is physically clustered with *tsaD*^5^. Interestingly, genomic context of these genes is variable across eukaryotes and prokaryotes indicating differences in their transcriptional organization and control mechanisms^3^. However, as yet, transcriptional organization and regulation of these genes have not been studied in any of the organisms where tRNA-A_37_-t^6^A transferase pathway is experimentally characterized. Knowing that TsaB and TsaE are essential as well as unique to bacteria, these proteins are regarded as promising antibacterial and inhibitor targets^3^,^4^,^6^,^7^. Therefore, an understanding of their genetic context, transcriptional organization and control, preferably in an organism-specific manner, is highly desirable^8^.

*M. tuberculosis* is the causative agent of Tuberculosis (TB), a major infectious disease of human. Since late 1980s, the disease has been undergoing a resurrection driven by a variety of changes in social, medical and economic factors, thereby leading to the rise of multiple drug resistant (MDR) and extensively drug resistant (XDR) strains of *M. tuberculosis*. This fastidious pathogen can survive hostile environments. Though not much studied, modified ribonucleosides in tRNA are now implicated in virulence and as growth rate determinants in pathogenic bacteria^9^. Therefore, it is plausible that the pathogenic mycobacteria may also employ RNA modifications to regulate translation and survive the human innate immunity. Recently, a chromatography-coupled mass spectrometric approach to identify and quantify ribonucleosides in *M. bovis* Bacille Calmette-Guérin (BCG), a species closely related to *M. tuberculosis*, confirmed the presence of t^6^A modification in mycobacteria^10^. Accordingly, the genes implicated in tRNA-A_37_-t^6^A biosynthesis are present in all mycobacterial species including *M. tuberculosis* (Supplementary Figure S1).

In *M. tuberculosis*, the homologs of tRNA-A_37_-t^6^A transferase machinery are present in a neighborhood where *tsaD* (Rv3419c), *tsaB* (Rv3421c) and *tsaE* (Rv3422c) are clustered together with *rimI* (Rv3420c) encoding an N-α-acetyltransferase and *alr* (Rv3423c) that codes for a well-characterized essential enzyme alanine racemase (Supplementary Figure S1). Presence of *rimI* in this genetic milieu is uniquely conserved in some genera like *Bacillus, Clostridium, Corynebacterium* and *Mycobacterium* (http://string-db.org)^4^,^8^. In a parallel study, our group has recently characterized RimI as a protein N-α-acetyltransferase having significantly relaxed substrate specificity, encompassing specificities of major eukaryotic NATs and mimicking NatE the closest^11^. Both functional and genetic context of RimI is yet little explored in bacteria, in general. Further, in *M. tuberculosis, tsaD* and *tsaB* are required for bacterial survival in primary murine macrophages, and essential for *in vitro* growth of bacteria on cholesterol^12^,^13^. Presence of transcripts of *tsaD* (Locus ML0379 in *M. leprae*) in the skin lesions of leprosy patients further emphasizes role of *tsaD* in pathogenicity of mycobacteria^14^. Similarly, *tsaE* is an essential gene according to Himar1-based transposon mutagenesis assays in H37Rv strain^15^. Alr catalyzes the synthesis of *d*-alanine that is needed for peptidoglycan biosynthesis^4^. Essentiality of *alr* for cellular viability is already known through knockout/knock down and transposon mutagenesis studies^13^,^15^,^16^. The cluster of genes from *alr to tsaD* (termed *gcp-alr* in TBDB, http://www.tbdb.org) is located immediately upstream of a previously characterized bicistronic operon *groESL1* (Rv3418c and Rv3417c) encoding essential chaperones^17^. Despite medical importance of TB pathogen, understanding of operon architectures and regulation thereof is very poor in *M. tuberculosis*. This fact is further corroborated by our comprehensive compilation of experimentally validated and published information about operons in *M. tuberculosis* until October 2015 (Supplementary Table S5)^18^. Therefore, for the reasons that: (a) most genes in this cluster are essential for survival or virulence of *M. tuberculosis*, (b) they exist in an interesting context with a protein N-α-acetyltransferase, (c) the transcriptional organization and regulation of tRNA-A_37_-t^6^A transferase pathway is not known in bacteria yet, and d) the tRNA-A_37_-t^6^A transferase machinery is differently organized across different bacterial species, we decided to investigate this genetic context at the transcriptional level.

## Results

### Homologs of *tsaD, tsaB and tsaE* are co-transcribed with *alr, rimI* and *groESL1* sub-operon in *M. tuberculosis*

**Figure 1:**
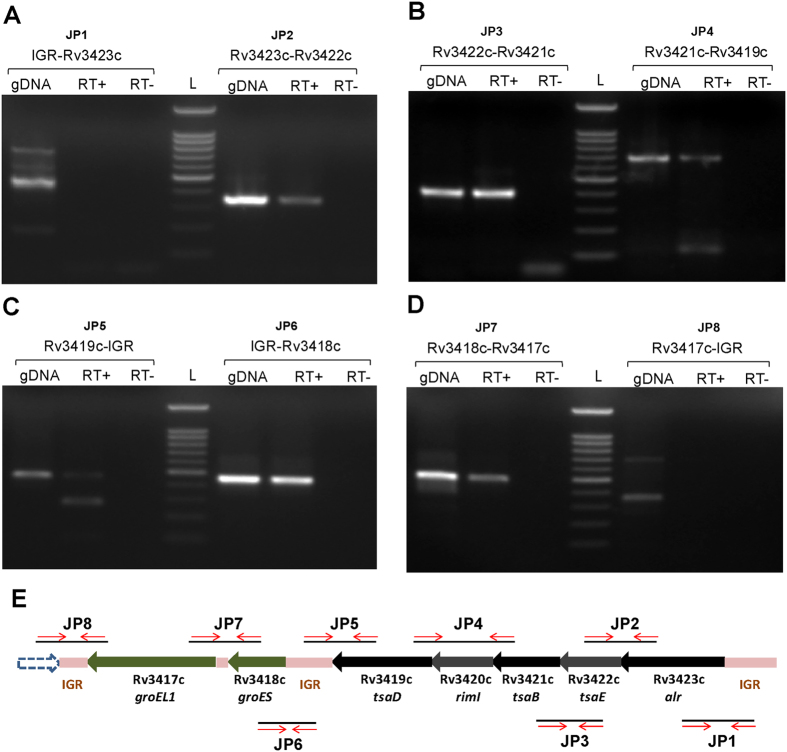
Cotranscription of *alr-gcp* gene cluster as determined by RT-PCR. **(A**–**D)** Total RNA from *M. tuberculosis* H37Rv in exponential phase was reverse transcribed and the cDNA thus made was used as template for PCR. Primers were designed in order to amplify the junctions between adjacent genes, shown above each gel. Each panel is constituted by three lanes – gDNA lane, which contains amplified product from genomic DNA and serves as positive control; RT+ lane, contains PCR product from cDNA; RT− lane, the negative control, represents the PCR product from possible gDNA present as contaminant in RNA preparation used for cDNA synthesis. **(E)** A schematic representation of the position of primers designed for cotranscription analysis of the gene cluster.

**Figure 2:**
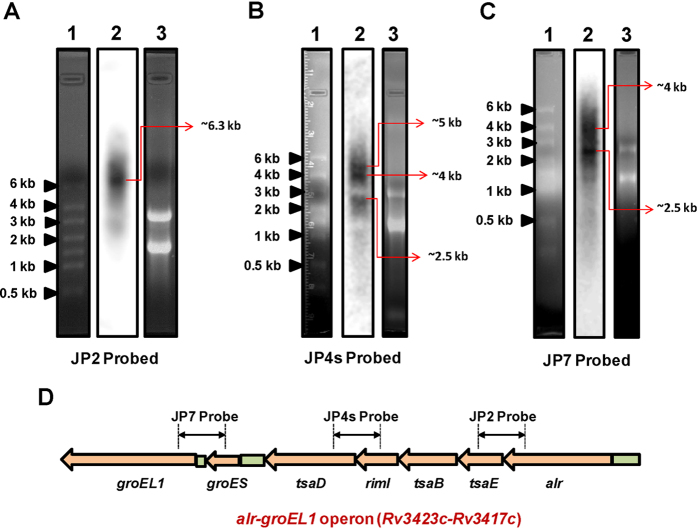
Multiple transcripts are produced from *alr-groEL1* operon. Northern blotting of *M. tuberculosis* H37Rv total RNA was performed. About 5 to 8 μg total RNA was resolved on formaldehyde agarose gel (1.2% in panel **A** and 1.5% in panels **B** and **C**) and transferred to nylon membrane. Blotted RNA was then separately probed with radiolabeled JP2 (panel **A**), JP4s (panel **B**) and JP7 (panel **C**) dsDNA probes. In each panel, lane 1 represents the RNA ladder, lane 2 is the blot and lane 3 shows the RNA sample. **(D)** Schematic representation of the *alr-groEL1* operon showing the regions used for probe generation.

A consecutive 4 bp overlap of stop and start codons of genes *alr* to *tsaD* conjectures a five-gene long operon organization that is predicted as *gcp-alr* operon in TBDB. To test this, we first verified the presence of the individual transcripts corresponding to *alr, tsaE, tsaB, rimI* and *tsaD* in the total RNA pool of *M. tuberculosis* H37Rv representing constitutive condition using gene specific primers. Extensive RT-PCR experiments using appropriate primers (Supplementary Table S1) corresponding to all possible junctions between genes of the *gcp-alr* cluster with suitable controls, helped us to ascertain the beginning of the ~6–6.5 kb transcript at *alr* and the end just after *groEL1* (Fig. 1; see also Supplementary Figure S2). These experiments conclusively confirmed the co-transcription of five genes, namely *alr, tsaE, tsaB, rimI, tsaD* along with *groEL1* and *groES*, in constitutive condition (Fig. 1). In order to determine the size of the longest cotranscript, northern blotting was performed on total RNA using a 300 bp dsDNA probe (radiolabeled JP2) encompassing the junction of *alr* and *tsaE* genes. A band was spotted on the blot at around ~6–6.5 kb, thereby providing the evidence of a transcription unit longer than those predicted by *in silico* methods (Fig. 2A). These results led to the conclusion that the transcript harbours all the seven genes with *alr* as the first while *groEL1* as the last gene. The *groES* and *groEL1* genes have already been shown to form an independent bicistronic *groESL1* operon with two TSSs annotated within the intergenic region between *tsaD* and g*roES*^17^. Our results go beyond *in silico* predictions and elucidate *alr-gcp* transcript and previously known, bicistronic *groESL1* transcript as products of larger heptacistronic *alr-groEL1* operon.

### Mapping the transcription start sites in the *alr-groEL1* operon

To identify transcription start sites (TSSs) in the *alr-groEL1* operon, 5′ RLM-RACE was performed on total RNA isolated from *M. tuberculosis* H37Rv culture under physiological conditions. As multiple transcripts originating from the operon were observed in northern experiments, and primer extension experiments were not conclusive (Supplementary Figure S4), we employed various gene-specific and nested gene-specific primers along with 5′ generacer primers to locate the possible TSSs in the operon. RACE reactions were compared with mock reactions lacking TAP treatment to facilitate the identification of unambiguous TSSs. Two transcription start sites were mapped in the operon- one at nucleotide G located at −263 position (3841684 genome position) relative to the *alr* initiation codon GTG while the other was at the nucleotide T located at −275 position (3838864 genome position) with respect to the *tsaD* initiation codon ATG (Supplementary Figure S3 and Table S2). Corresponding to these start sites, −10 hexamer sequences, namely TAGGTT and TACGCT are also identified upstream of *alr* and *tsaD* genes, respectively (Supplementary Table S3). The sequences of −10 hexamers here conform to the mycobacterial promoter consensus (TANNNT at −10 box) as defined by Cortes *et al*.^19^. In Supplementary Table S2, we have tabulated all the TSSs located within a*lr-groEL1* operon that are identified by us and or others, using different methods in independent studies^19^,^20^.

### Multiple transcripts suggest intricate operon architecture

With *groESL1* operon already annotated^17^, it was anticipated that at least two differently sized transcripts should originate from this newly identified heptacistronic *alr-groEL1* operon. To authenticate the same, northern blotting was performed using two new radiolabeled probes, namely JP4s (300 bp) and JP7 (500 bp) as explained in Fig. 2B and C, in addition to previously employed probe JP2 (Fig. 2A). Multiple bands were obtained on the blot representing possible transcripts originating from the *alr-groEL1* operon. The probes JP2, JP4s and JP7 together, broadly cover the entire operon and provide an overall information about transcripts arising from the *alr-groEL1* operon. In this experiment, we could successfully detect the smaller bicistronic transcript (~2–2.5 kb; Fig. 2C) originating from the *groESL1* sub-operon. Presence of transcripts of different lengths strongly hinted at the presence of more than one regulatory element (promoters, terminators) in the heptacistronic operon.

### Six different promoter elements of varying strength control the operon

**Figure 3:**
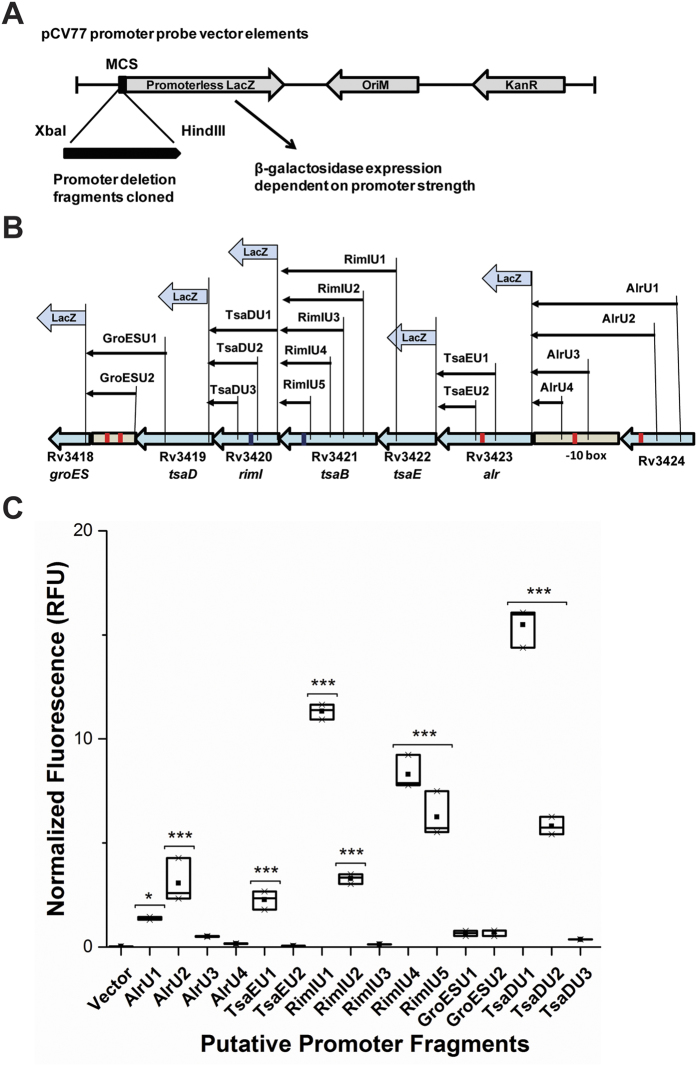
Multiple promoters control the transcription of *alr-groEL1* operon. **(A)** pCV77 vector design and cloning of promoter deletion fragments. **(B)** A schematic representation of promoter deletion fragments design. **(C)** Relative strength/activity of putative promoter fragments from *alr-groEL1* operon as determined by normalized fluorescence intensity (in RFU) which is a measure of β-galactosidase expression. *M. smegmatis* mc^2^155 was transformed with promoter plasmids (fragments cloned in pCV77 promoter probe vector) and grown to approx. OD_600_ 1.0. The cells were then sonicated for 3 min 15 s and β-galactosidase activities measured using FluoReporter *lacZ*/Galactosidase Quantitation Kit. Fluorescence intensity values were normalized against protein concentration and a given reference standard. Box chart has been plotted to show median (represented by horizontal line), mean (represented by black filled box) interquartile range (represented by box), and maximum and minimum values (represented by whiskers). The results shown were generated from three independent experiments, each with three technical replicates. The asterisks shown above each box plot represent the statistically significant difference from the vector control and are derived from *p* values with *for *p* < 0.05, **for *p* < 0.01 and ***for *p* < 0.001. Statistics was performed using one-way ANOVA with Dunnett’s multiple comparison test.

The promoter elements and their architecture in *M. tuberculosis* are variable and not always obvious upon sequence inspection using *in silico* tools (ex. BPROM) that are tailored for organisms like *E. coli*. Manual inspection of the genomic sequence of the operon in accordance with available literature^18^,^19^,^20^,^21^ helped us to identify possible promoter elements in the *alr-groEL1* operon. After making a careful judgment from all predictions and northern blot results put together, series of genomic fragments across the length of the operon were cloned in an *in vivo* promoter assay system (Fig. 3A and Table 1). The resulting clones were transformed into *M. smegmatis* cells and assayed for β-galactosidase activity using flourescent substrate. Among the promoter fragments upstream of *alr*, the minimal promoter was identified as P_*alrU2*_ that contains mycobacterial consensus sequence TAGGTT at −10 position.With fragment larger than P_*alrU2*_ i.e. P_*alrU1*_, a decrease in activity was observed indicating a possibility of repressive motifs within the 466–584 bp region upstream of *alr* gene (Fig. 3B). It has earlier been studied that repression elements in the DNA region upstream of a promoter can lead to irregularities in the activity of the deletion fragments of that promoter^22^. Similarly, three more promoters were detected in the coding regions of *alr, tsaB* and *rimI* genes that are upstream of *tsaE, rimI* and *tsaD*, respectively. The minimal promoters identified are designated as P_*tsaEU1*_, P_*rimIU5*_ and P_*tsaDU2*_, respectively. While a conserved −10 hexamer sequence, namely TAYgAT (Y is pyrimidine) is found in mycobacterial promoters, little homology exists among −35 sequences indicating the absence of a defined −35 sequence and its dispensability for promoter function^23^. However, a conserved −10 motif TANNNT, was recently found to be associated with most of the primary TSSs in mycobacteria^19^. Accordingly, among the new promoters identified in the *alr-groEL1* operon and summarized in Supplementary Table S3, three, namely P_*alrU2*_, P_*rimIU5*_ and P_*tsaDU2*_ harbour mycobacterial canonical promoter elements. This consensus is slightly relaxed compared to the TATAAT consensus found in *E. coli* and several other bacteria. About half of the TANNNT promoters in *M. tuberculosis* have been shown to be associated with a 3 bp extended −10 motif responsible for the upsurge in the activity of the concerned promoter^19^. We detected this 3 bp consensus motif, [G/C][A/G]N (SRN), in the promoters located upstream of *tsaD* and *alr* genes. P_*tsaDU1*_ also exhibited the highest β-galactosidase activity (at least 23-fold higher than that of the heat shock promoter P_g*roESU2*_). The activity displayed by the minimal promoter P_*tsaDU2*_ (with extended consensus motif) was comparable to that seen in case of minimal P_*rimIU5*_ promoter (without extended motif). In the COG category of RNA processing and modification genes, the proportion of leaderless genes (having very short or missing 5′-UTRs) is much higher than in other categories, in bacteria, in general^24^. Though the genes in *alr-groEL1* operon belong to RNA processing/modification category of genes, yet leaderless architecture is completely absent here.

### An I-shaped hairpin-like *cis*-regulatory element is integral to the operon

**Figure 4:**
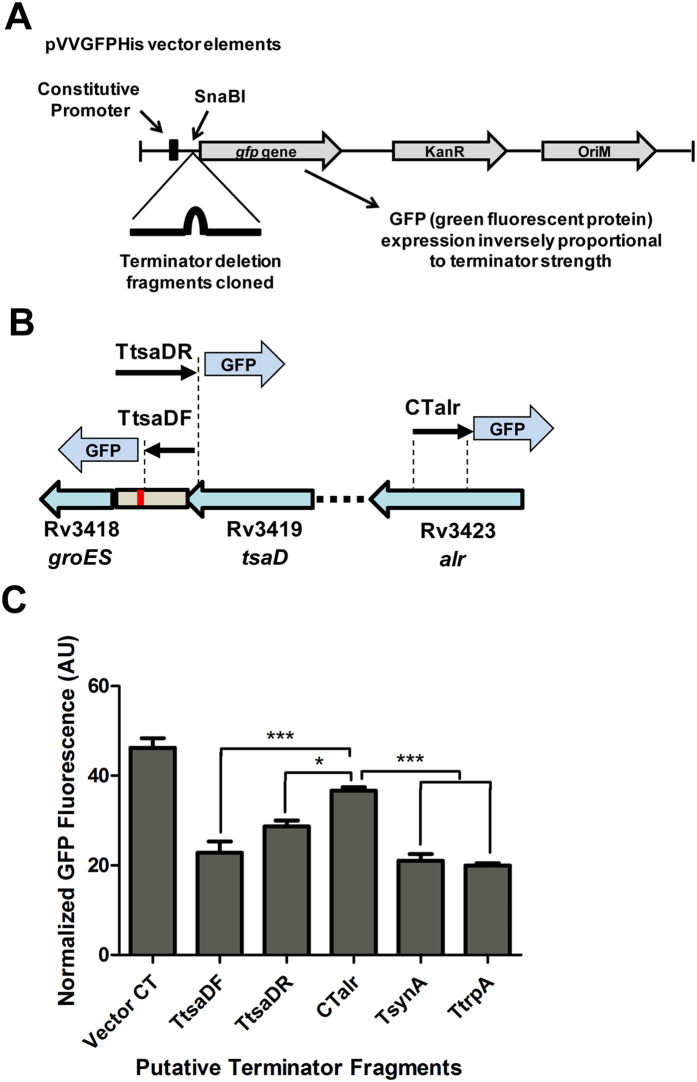
The *alr-groEL1* operon harbors an I-shaped hairpin-like *cis*-regulatory element, T_*tsaD*_. **(A)** Basic elements of pVVGFPHis vector used for cloning. **(B)** Graphical representation of tested fragments for termination efficiency. **(C)** Measurement of *in vivo* GFP fluorescence plotted as comparative reduction indicating termination strength of tested terminator-like fragments. *M. smegmatis* mc^2^155 cells were transformed with various putative terminator fragments cloned downstream of constitutive promoter of *gfp* gene in pVVGFPHis vector. The vector with no intervening foreign sequence between the promoter and GFP coding region, served as the negative control. Two synthetic terminators, T_*synA*_ and T_*trpA*_, provided for positive controls for transcription termination. Log phase cultures were assayed for GFP fluorescence in triplicate in black 96-well microplate. The fluorescence was measured at 395 nm excitation and 509 nm emission wavelengths. The results shown are means ± SEM of three independent experiments, each accomplished with three technical replicates. One-way ANOVA with Dunnett’s multiple comparison test was employed to determine *p* values.

Promoters alone did not explain northern blot results completely (Fig. 2). Since *groESL1* operon has already been described^17^, its upstream sequence was analyzed for the presence of internal terminators/ pause signals, if any, using GeSTer algorithm (Genome Scanner for Terminators)^25^. The tool predicted a non-canonical I-shaped, factor-independent terminator T_*tsaD*_, downstream of *tsaD* gene. A series of fragments harbouring predicted and known reference terminators were cloned between the coding sequence of GFP and its promoter in the pVVGFPHis vector (Table 1). Reduction in GFP fluorescence was measured to decipher termination efficiencies (Fig. 4A and B). Significant reduction in GFP fluorescence as compared to CT*alr* control confirmed reliable *in vivo* transcriptional regulation by T_*tsaD*_ (Fig. 4C). A typical *E. coli* intrinsic terminator has a U-trail post palindromic stem loop/ hairpin. Non-canonical I- shaped intrinsic hairpins lack a U-trail (Supplementary Table S3). Such hairpins are fairly common among GC rich mycobacterial genomes. As the sequence of T_*tsaD*_ lacks any U-tract and reversing the orientation of the hairpin sequence (T_*tsaD*_R) didn’t lead to any significant reduction in the fluorescence of the gene downstream of the fragment (Fig. 4C), the element is akin to an I-shaped terminator in accordance with the description provided by Unniraman *et al*.^26^.

Low or partial *in vivo* termination efficiency in such I-shaped hairpins is also known^25^. Recently, it was demonstrated that *M. bovis* RNA polymerase requires termination-stimulating activity of mycobacterial NusG and a U-tract, which may be imperfect for termination at intrinsic terminators *in vitro*. It was also demonstrated that intrinsic terminators lacking imperfect or perfect U-tract are not terminated by *M. bovis* RNA polymerase^27^. However, in our experiments, the *in vivo* termination efficiencies of T_*tsaD*_, T_*synA*_ and T_*trpA*_ (previously *in vitro* validated terminators are explained in Table 1 and ref. 27) were found to be comparable (Fig. 4C). Therefore, in view of the above discussed contradictory evidences about the functionality of I-shaped terminators *in vivo*^26^,^27^,^28^, T_*tsaD*_ is defined as an I-shaped hairpin-like *cis*-regulatory element that might bring about reduction in transcription by acting as a pause signal and facilitating fraying of the elongation complex or by enabling factor-dependent termination after slowing down the complex *in vivo*^29^ or might influence transcriptional/post-transcriptional regulation of downstream genes by some other mechanism. Recently, a unique branching structure (with a trailing U-tract) was shown to function as an intrinsic terminator in *Enterococcus faecalis*^30^, sustaining the suggestion that greater flexibility might exist in the design of intrinsic terminator-type regulatory elements than has been contemplated so far.

### Multiple regulatory elements orchestrate a complex transcriptional profile of the operon under stress

**Figure 5:**
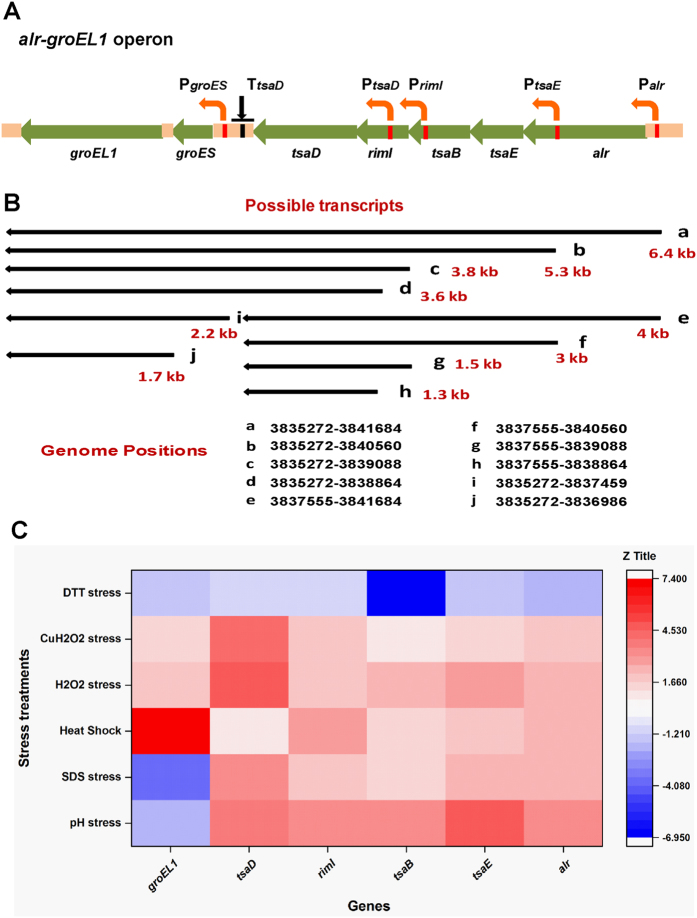
Relative expression of the constituent genes of *alr-groEL1* operon under various stress conditions. **(A)** A schematic representation of the positions of promoters (indicated by red bars) and possible pause signal/ transcription regulatory elements (represented by black bars). **(B)** The *alr-groEL1* multi-transcript map representing a probable outcome of previous experiments. Genome position for each transcript is indicated to estimate its approximate size. **(C)** A heatmap with colour variations referring to the changes in expression levels of the individual genes. Colour scale is represented by *z* title. Colour approaching red denotes upregulation while downregulation is represented by increasing blue colour. RT-qPCR was done on total RNA from *M. tuberculosis* H37Rv subjected to various stresses that include heat shock at 42 °C, SDS (0.1%), pH 4.5, H_2_O_2_ (5 mM), cumene hydroperoxide (5 mM), DTT (1 mM). In all cases, the bacteria were kept under stress for approx. 2 h except for heat shock which was given for 30 min. The results (fold change observed) were normalized to the expression of the 16S rRNA reference gene. The results shown are the representative of three independent experiments.

Multiple transcripts originating from a single operon, presence of multiple promoters or terminators and a sub-operonic architecture (with more than one independent control elements) provided enough clues about a complex yet fine mode of regulation of *alr-groEL1* operon^31^ (Fig. 5A). Transcripts of various sizes as detected on blots were aligned with the operon in all possible combinations and corroborated with other experiments to arrive at a logical map as represented in Fig. 5B. Keeping the pathogen biology and human physiology in consideration, a quantitative transcriptional response profile of individual genes in the operon was generated under heat shock, low pH stress (pH 4.5), SDS stress (0.1% SDS), oxidative stress (5 mM H_2_O_2_, 5 mM cumene hydroperoxide) and reductive stress (1 mM DTT) to obtain a broader picture of involvement of these genes in stress adaptation in *M. tuberculosis*^32^,^33^. Results of quantitative RT-PCR of RNA isolated from bacterial cultures subjected to different stresses are summarized in Supplementary Table S4 and presented as a heat map in Fig. 5C (individual stress responses are plotted in Supplementary Figure S5). The genes of the operon were upregulated under majority of the conditions studied except for reductive (DTT) stress. The *tsaD* and *alr* genes were most notably induced by the stresses. Under detergent and oxidative stresses, significant upregulation (~3.5-fold) was observed for *tsaD* gene while under pH stress, all genes except *groEL1* were induced. Heat shock experiments carried out at 42 °C in our study led to ~7-fold upregulation (measured through RT-qPCR) of *groESL1* operon while all other genes barring *tsaD* gene of the cluster, were slightly upregulated. Similarly, Aravindhan *et al*.^17^ had also reported 4-fold upregulation (measured through primer extension) of *groESL1* sub-operon upon heat shock^17^. Interestingly, *groES* was found about 9-fold upregulated in Δ*hspR*Δ*hrcA* strain of mycobacteria (measured through microarray) under constitutive condition previously, implying a role for HspR and HrcA in represson of *groESL1* sub-operon under constitutive condition^34^. This further reflects the significance of sub-operonic architecture where regulation of sub-operon is distinct from the main operon/upstream transcripts in stress especially during heat shock, pH and SDS stresses in *M. tuberculosis*. The heat map also highlights the discrete expression profile of *tsaD* gene under three of the stress conditions, namely pH, detergent and oxidative stresses. This expression pattern of *tsaD* can be explained by the presence of a strong upstream promoter, P_*tsaDU1*_ and an I-shaped hairpin-like *cis*-regulatory element, T_*tsaD*_ downstream of the gene. Heat map generated from the quantitative RT-PCR of operon genes as well as that of intergeneic regions of the operon (see Supplementary Figure S6) corroborate subtle fine-tuning that these multiple regulatory elements could bring about to the levels of the individual transcripts or cotranscripts in the cellular milieu.

## Discussion

The tRNA-A_37_-t^6^A transferase machinery consists of universally conserved protein TsaD, and proteins specific to bacteria, namely TsaB and TsaE^2^,^3^. Physical clustering of the encoding genes is observed in the genomes of several bacteria in different contexts, yet no experimental data is available on transcriptional organization and regulation of such an important and conserved pathway except for *Neisseria gonorrhoeae* wherein *tsaD* is shown to be co-regulated with cytochrome genes^35^. In this study, we have characterized a new multicistronic *alr-groEL1* operon that is involved in the transcriptional regulation of *tsaD, tsaB* and *tsaE* homologs in *M. tuberculosis*, a persistent human pathogen. The operon is, in fact, composed of two sub-operons: *alr-tsaD* and *groESL1*, both having independent control elements like promoters and terminators. Position of a newly identified internal I-shaped hairpin-like *cis*-regulatory element, T_*tsaD*_ has also been defined. Occurrence and importance of such sub-operon architecture in transcriptional regulation has previously been analyzed in *Bacillus subtilis* and *E. coli*^31^. The *alr- groEL1* operon is one of the few experimentally characterized operons where sub-operon architecture exists within the main operon, in *M. tuberculosis* (as per the information compiled in Supplementary Table S5). The sub-operonic architecture seems physiologically relevant as seen in case of heat shock, SDS and pH stress conditions where suboperon is distinctly regulated vis-à-vis main operon (Fig. 5). Complex transcriptional regulation is a characteristic feature of *M. tuberculosis* as observed in case of DNA gyrase bicistronic operon that is regulated by alternative internal promoters and competing promoters on the opposite strand^36^, and *recXA* operon which is controlled by multiple promoters for single gene^37^, and others listed in Supplementary Table S5. The case of *alr-groEL1* operon is interesting in the sense that it contains a large number of *cis*-regulatory elements, namely promoters and terminator-like *cis*-regulatory element. Whereas studies on identifying promoters and transcriptional factors are aplenty, terminators and similar *cis*-regulatory transcriptional elements are not much studied in *M. tuberculosis* (Supplementary Table S5). As seen in the case of *alr-groEL1* operon, careful analysis of such *cis*-regulatory elements is essential for unambiguous interpretation and understanding of the operon architecture in *M. tuberculosis*. Using our experimental evidence and tapping the relevant and corroborating information made available recently by others^19^ and as cited above, we have created a comprehensive map of *alr-groEL1* operon that summarizes the sub-operonic architecture generating multiple transcripts, complex co-transcription and co-regulation of essential putative t^6^A transferase machinery, chaperones, cell wall biosynthesis gene *alr* and an N-α-acetyltransferase in *M. tuberculosis* (Fig. 5 and Supplementary Figure S1; see also Supplementary Table S3 for detailed description of individual elements). The extensive information about 40 experimentally characterized operon structures in *M. tuberculosis* (see Supplementary Table S5) along with the findings of this manuscript can serve as ready information for bioinformatics analysis/ relevant tool development.

In parallel, we could also identify P_*tsaDU1*_ as ~23-fold and P_*rimIU1*_ as ~17-fold stronger promoters than the heat shock promoter P_g*roESU2*_ under constitutive condition in mycobacterial expression system. Characterization of the *tsaDU1* and *rimIU1* promoter fragments in this study provides for valuable tools for the expression of genes in mycobacteria.

The stress-induced mosaic transcriptional profile of individual genes depicted in heat map corroborates well with the complex architecture of the operon harboring multiple regulatory elements. The heat map also indicates fluid dynamics of bacterial operon involved in multiple stress adaptations, wherein multiple regulatory elements can give rise to variable transcripts under different environmental conditions (Fig. 5C and Supplementary Figure S6). While it could also be a mechanism of energy and resource conservation under non-optimal survival conditions, such intricate regulatory designs may be important for accommodating the dynamic changes that happen within or around the cell. Such differences may play an important role in the evolution of a gene order across genomes^31^. Since the t^6^A modification is known in *M. bovis* and levels of t^6^A modification are implicated in regulating gene expression by controlling selective translation in codon-biased manner as the organism goes through hypoxia-induced latency and resuscitation later on^38^,^39^,^40^,^41^, we revisited the heat map summarizing the results of quantitative RT-PCR of *alr-groEL1* operon. Indeed, during oxidative stress, almost all genes of the operon are upregulated, including *tsaD* (an essential component in the threonylcarbomyl tRNA synthesis). A general upregulation of *tsaD, tsaB* and *tsaE* genes, therefore, might be a prerequisite for t^6^A modification during oxidative stress. Accordingly, this study opens up further interest towards understanding the transcriptional and stress regulation of t^6^A transferase enzymes in bacteria and in pathogenic *M. tuberculosis,* specifically.

Using mass spectrometry-based enzyme assays, we had previously identified an enzyme-substrate relationship between RimI (N-α-acetyltransferase) and GroES, GroEL1 and TsaD proteins as substrates wherein N-terminus of these proteins is acetylated by RimI *in vitro*^11^. With the help of an *in vivo* mycobacterial protein fragment complementation (MPFC) assay conducted in *M. smegmatis*, protein-protein interactions between RimI and GroES, RimI and TsaD, and TsaD and TsaE proteins are also known to us^11^. Although functions of the 10 kDa mycobacterial GroES/Cpn10 protein are not known in detail, GroES is reported to be N-α-acetylated in *M. tuberculosis* proteome^42^. Therefore, co-transcription of *groESL1* and *rimI* under *alr-groEL1* operon supports the previously identified involvement of RimI in the functioning of the chaperone protein. Further, a weak *in vitro* interaction between a recombinant T18-RimI fusion protein and T25-TsaB fusion protein is reported in *E. coli*, previously^4^. Taking into account the conservation of *tsaB* and *rimI* synteny across many bacterial species (http://string-db.org) including pathogenic *Neisserial* and *Mycobacterial* species where a *tsaB-rimI* gene fusion is witnessed (ex. *M. leprae*), results from this study and our previously published results^11^, the involvement of RimI in TsaD, TsaB and TsaE functioning is anticipated. The *alr-groEL1* operon thus provides a well-defined transcriptional context to study hitherto unexplored regulation of RimI/ protein N-α-acetylation as well as involvement of RimI/protein acetylation in tRN A-A_37_-t^6^A modification pathway, in *M. tuberculosis.*

## Materials and Methods

### Bacterial strains and culturing

The bacterial strains and plasmids used in this study are listed in Table 1. Middlebrook 7H9 broth supplemented with 10% ADC (albumin and dextrose complex), 0.2% glycerol and 0.1% Tween 80, was used for culturing *M. tuberculosis* H37Rv and *M. smegmatis* mc^2^155. Middlebrook 7H11 agar supplemented with 10% OADC (ADC with oleic acid) and 0.5% glycerol, was used for transformation and growth of *M. smegmatis* mc^2^155. The *E. coli* cells were grown in Luria-Bertani (LB) medium. *M. smegmatis* mc^2^155 was grown on X-Gal- and kanamycin-containing 7H11 agar plates for β-galactosidase reporter assays. About 800 μg X-Gal was spread onto each 7H11 plate. *E. coli* XL1-Blue and TOP10 strains were used for transformation. Kanamycin was used at final concentrations of 50 μg/ml for *E. coli* and 30 μg/ml for *M. smegmatis.* All experiments were performed under constitutive condition unless mentioned otherwise.

### RNA Extraction

*M. tuberculosis* H37Rv cultures grown at 37 °C until log phase were first treated with 35 ml GTC Buffer (5 M Guanidine thiocyanate, 0.5% Tween-80, 1% β-Mercaptoethanol) per 15 ml of culture followed by pelleting the cells. The cells were processed immediately for extraction of total RNA using FastRNA^R^ Blue Kit (MP Biochemicals) as per the manufacturer’s instructions, where chloroform phase separation and isopropanol precipitation were repeated twice to ensure good quality. Residual DNA was removed by treating approx. 40 μg RNA with 4 μl DNase I, RNase-free (Ambion) in the given reaction buffer in a total volume of 80 μl at 37 °C for 30 min. DNase I treated samples were further purified using Qiagen Rneasy MinElute Cleanup kit and DNase I treatment was repeated again to ensure proper removal of genomic DNA. Finally, DNase I was inactivated at 75 °C for 5 min in presence of 5 mM EDTA, and RNA was used for RT-PCR and RT-qPCR.

### Reverse transcriptase PCR (RT-PCR)

First-strand cDNA synthesis was carried out using RevertAid M-MuLV Reverse Transcriptase kit (Fermentas) as per the instructions of the manufacturer. Nearly 1 μg purified RNA acting as a template, was incubated with random hexamers and denatured at 65 °C for 5 min followed by snap chilling. Other reaction components were subsequently added to the tube in 20 μl final volume. The tube was incubated in a thermal cycler at 25 °C for 5 min, 45 °C for 1 h and 70 °C for 5 min, consecutively. Along with the cDNA reaction, a negative control reaction, termed RT-, was also included from which RT enzyme was omitted to rule out the presence of any contaminating genomic DNA. PCR reactions were performed with 1 μl template from each reaction (cDNA rxn, RT- rxn). In parallel with these reactions, another positive control PCR reaction was set up from gDNA template. The PCR products were loaded on an agarose gel followed by visualization on a UV transilluminator.

### Northern blotting

Northern analysis was performed with *M. tuberculosis* H37Rv total RNA as per the standard protocol^43^. Briefly, 5 to 8 μg of total RNA isolated from log phase cultures was resolved on 1.2% or 1.5% agarose gel containing 6% formaldehyde. RNA was transferred to Amersham Hybond-N + nylon membrane (GE Healthcare) overnight using upward capillary transfer. It was then cross-linked to membrane by exposure to UV and incubated with denatured radiolabeled probe after pre-hybridization. [α-P^32^] dCTP-labeled DNA probes were generated near the 5′ and 3′ ends of the operon. Hybridization was done overnight at 42 °C, followed by several membrane washes. Finally, the blot was exposed to phosphorimager screen which was then scanned using phosphorimager ImageQuant to visualize the hybridization signals.

### 5′ RLM-RACE

RNA ligase-mediated rapid amplification of 5′ cDNA ends (5′′ RLM-RACE) was carried out using GeneRacer kit (Invitrogen) and total RNA from *M. tuberculosis*. Manufacturer’s instructions were followed with some modifications. Briefly, 5 μg total RNA was column purified after DNase-I (Ambion) digestion and then treated with calf intestinal phosphatase (CIP) and tobacco acid pyrophosphatase (TAP) sequentially. CIP was used to dephosphorylate truncated mRNAs and non-mRNAs while TAP was employed to generate a 5′ monophosphate on intact mRNAs by removing the terminal pyrophosphate group. Next, the RNA was ligated to 5′ RACE adaptor (RNA oligo) and reverse-transcribed to cDNA using random hexamers and Superscript III Reverse Transcriptase at 50 °C for 1 h. To distinguish between processed and primary transcripts, a mock reaction (TAP-) was set up in parallel that was kept from TAP treatment. The cDNA was amplified with Phusion High Fidelity DNA polymerase (NEB) using GeneRacer 5′ primer (complimentary to a part of RNA adapter sequence) in combination with gene-specific reverse (GSR) primers, namely Rv23R (5′-CCGACATTCTCCCAGAACCG) and TsaDUR (5′-GTTCAAGCTTGACTGTCGTCATGACGGGTCC). Touch-down PCR protocol was followed for initial amplification from cDNA as: (30 sec at 98 °C) × 1 cycle, (10 sec at 98 °C, 1 min at 72 °C) × 5 cycles, (10 sec at 98 °C, 1 min at 70 °C) × 5 cycles, (10 sec at 98 °C, 30 sec at 68 °C, 1 min at 72 °C) and finally 5 min at 72 °C. Later, nested GSR primers (AlrUR, 5′-GTTCAAGCTTCTGAGCAGTATTCCGGCCTG; Qt20R, 5′-TCATTATCGGTGCGGACCTCCA) were used to generate specific products from initial PCR reactions (Supplementary Table S1). The PCR products were gel-purified and cloned in pJET1.2/blunt vector using CloneJET PCR cloning kit (Fermentas), and sequenced with pJET1.2 forward and reverse sequencing primers.

### *In vivo* promoter activity assay

Putative promoter sequences were PCR amplified from *M. tuberculosis* H37Rv genomic DNA using Phusion High Fidelity DNA Polymerase (Finnzymes)/ KOD Hot Start DNA polymerase (Novagen) using appropriate primers (Supplementary Table S1). The PCR products were digested with XbaI and HindIII enzymes and cloned ahead of *lacZ* gene in promoter-less shuttle vector pCV77 in *E. coli* creating series of promotor fragment clones for *in vivo* analysis as shown in Table 1. *M. smegmatis* transformants containing individual clones were selected on X-gal-containing 7H11 agar plates. Independent transformants were grown in ~5 ml 7H9 broth until an OD_600_ of 1. Cells were pelleted down, resuspended in 300 μl lysis buffer (1X PBS, protease inhibitor cocktail) and lysed by sonication for 3 min 15 s with 15 s pulse and 15 s rest at 30% amplitude using Sonics Vibra-Cell^TM^ VCX500 with 3 mm stepped microtip. The supernatant from the lysate of each transformant was analysed for β-galactosidase activity using FluoReporter^R^ *lacZ*/Galactosidase Quantitation Kit (Molecular Probes) as per given protocol. The fluorescence intensity of the samples was measured in triplicate in Nunc^TM^ black 96-well plates using TECAN Infinite^R^ M200 PRO microplate reader. The excitation and emission wavelengths used were 390 nm and 460 nm, respectively.

### *In vivo* transcriptional terminator activity assay

Selected putative terminator sequences as listed in Table 1 were PCR amplified as above (using primers listed in Supplementary Table S1), cloned in SnaBI site after GFP promoter in pVVGFPHis vector and transformed in *M. smegmatis* mc^2^155. Cloned terminator fragments include: T_*tsaD*_F: 123 bp downstream region of *tsaD*- forward sequence; T_*tsaD*_R: 275 bp downstream region of *tsaD*- reverse sequence; CT*alr*: 156 bp coding region of *alr*; T_*synA*_: 40 bp synthetic hairpin sequence with a U-tract; and T_*trpA*_: 27 bp synthetic hairpin sequence with a U-tract (based on *E. coli* tryptophan operon terminator). Two synthetic terminators, T_*synA*_ and T_*trpA*_, were included to serve as positive controls for transcription termination. Three colonies of each terminator fragment were streaked onto 7H11 agar plate with kanamycin antibiotic (30 μg/ml) and then inoculated in 2 ml 7H9 broth. The cultures were grown for 3 days in a shaking incubator set at 37 °C and 200 rpm, and then sub-cultured in fresh 7H9 media with 5% inoculums. At an OD_600_ of 0.8, culture volumes of 120 μl were loaded into the black 96-well microplate in triplicate. GFP fluorescence intensity was measured at 395 nm excitation and 509 nm emission wavelengths in Nunc^TM^ black 96-well plates using TECAN Infinite^R^ M200 PRO microplate reader. The fluorescence intensity values were normalized against OD_600_ values.

### RT-qPCR (quantitative PCR)

*M. tuberculosis* H37Rv cultures were grown until log phase. Cells were washed with 1X PBS buffer and replenished with fresh 7H9 media containing 5 mM H_2_O_2_ or 5 mM cumene hydroperoxide to generate oxidative stress condition, 0.1% SDS to generate detergent stress condition, 1 mM DTT to generate reducing stress condition. Similarly, for acid stress treatment, log phase grown cells were replenished with fresh 7H9 media having pH 4.5. The freshly replenished cultures as well as appropriate controls were subjected to the respective stress condition for 2 h at 37 °C at 100 rpm. For heat shock, log phase cultures were first replenished with fresh 7H9 media and then transferred to 42 °C for 30 min.

Total RNA was extracted from all stress-treated *M. tuberculosis* cultures as well as control cultures as described above. The cDNA made with random hexamers was used as a template and qPCR was performed with gene-specific primers using Maxima SYBR Green qPCR Master Mix (Fermentas). Threshold cycle (*C*_*T*_) of the internal 16S rRNA control was utilized for the normalization of the calculated *C*_*T*_ value. The qPCR results were analyzed using REST 2009 software version 1 (see legend of Supplementary Figure S5).

## Additional Information

**How to cite this article**: Bhat, A. H. *et al. The* *alr-groEL1* operon in *Mycobacterium tuberculosis*: an interplay of multiple regulatory elements. *Sci. Rep.* **7**, 43772; doi: 10.1038/srep43772 (2017).

**Publisher's note:** Springer Nature remains neutral with regard to jurisdictional claims in published maps and institutional affiliations.

## Supplementary Material

Supplemental Information

Supplementary Table S5

## Figures and Tables

**Table 1:** Bacterial strains and plasmids used in this study.

Bacterial strain or plasmid	Description	Source or reference
*Escherichia coli* TOP10	F^−^ *mcrA ∆*(*mrr-hsdRMS*-*mcrBC*) ϕ80*lacZ∆*M15 *∆lacX74 recA1 araD139 ∆*(*ara leu*)*7697 galU galK rpsL (StrR*) *endA1 nupG*	Lab Collection
*Escherichia coli* XL-1 Blue MRA	*∆(mcrA) 183 ∆(mcrCB-hsdSMR-mrr) 173 endA1 supE44 thi-1 recA1 gyrA96 relA1 lac*	Stratagene
*Mycobacterium smegmatis* mc^2^155	High-transformation mutant of *M. smegmatis* ATCC 607	ATCC 700084
*Mycobacterium tuberculosis* H37Rv	Laboratory strain	ATCC 27294
pCV77 plasmid	Replicating *E. coli*-*Mycobacteria* shuttle vector with promoterless LacZ gene, Kan^R^ due to *aph* gene	MedImmune
pCVAlrU1	584 bp (3841420–3842004 genomic region) upstream *alr* cloned in pCV77 with AlrU1F and R primers	This study
pCVAlrU2	466 bp (3841420–3841886 genomic region) upstream *alr* cloned in pCV77 with AlrU2F and R primers	This study
pCVAlrU3	188 bp (3841420–3841608 genomic region) upstream *alr* cloned in pCV77 with AlrU3F and R primers	This study
pCVAlrU4	79 bp (3841420–3841499 genomic region) upstream *alr* cloned in pCV77 with AlrU4F and R primers	This study
pCVTsaEU1	424 bp (3840197–3840621 genomic region) upstream Rv3422c cloned in pCV77 with TsaEU1F and R primers	This study
pCVTsaEU2	338 bp (3840197–3840535 genomic region) upstream Rv3422c cloned in pCV77 with TsaEU2F and R primers	This study
pCVRimIU1	725 bp (3839062–3839787 genomic region) upstream Rv3420c cloned in pCV77 with RimIU1F and R primers	This study
pCVRimIU2	483 bp (3839062–3839545 genomic region) upstream Rv3420c cloned in pCV77 with RimIU2F and R primers	This study
pCVRimIU3	335 bp (3839062–3839397 genomic region) upstream Rv3420c cloned in pCV77 with RimIU3F and R primers	This study
pCVRimIU4	262 bp (3839062–3839324 genomic region) upstream Rv3420c cloned in pCV77 with RimIU4F and R primers	This study
pCVRimIU5	120 bp (3839062–3839182 genomic region) upstream Rv3420c cloned in pCV77 with RimIU5F and R primers	This study
pCVGroESU1	926 bp (3837288–3838214 genomic region) upstream Rv3418c cloned in pCV77 with GroESU1F and R primers	This study
pCVGroESU2	277 bp (3837288–3837565 genomic region) upstream Rv3418c cloned in pCV77 with GroESU2F and R primers	This study
pCVTsaDU1	500 bp (3838589–3839089 genomic region) Rv3420c gene cloned in pCV77 with TsaDU1F and R primers	This study
pCVTsaDU2	310 bp (3838589–3838899 genomic region) upstream Rv3419c cloned in pCV77 with TsaDU2F and R primers	This study
pCVTsaDU3	140 bp (3838589–3838729 genomic region) upstream Rv3419c cloned in pCV77 with TsaDU3F and R primers	This study
pVVGFPHis plasmid	Mycobacterial constitutive expression shuttle vector containing *gfp* and Kan^R^ genes. Developed from pVV16 plasmid.	BEI Resources, NIAID, NIH
pVVTsaDF	123 bp (3837555–3837432 genomic region) downstream of Rv3419c cloned in pVVGFPHis vector	This study
pVVTtsaDR	275 bp (3837555–3837280 genomic region) downstream of Rv3419c cloned in pVVGFPHis vector in reverse orientation.	This study
pVVCTalr	156 bp (3840806–3840962 genomic region) coding region of Rv3423c cloned in pVVGFPHis vector.	This study
pVVTsynA	40 bp synthetic hairpin sequence with a U-tract cloned in pVVGFPHis vector	This study
pVVTtrpA	27 bp synthetic hairpin sequence with a U-tract cloned in pVVGFPHis vector (based on *E. coli* tryptophan operon terminator)	This study
