# Supplementary material for: The alr-groEL1 operon in Mycobacterium tuberculosis: an interplay of multiple regulatory elements
Source: Sci Rep. 2017 Mar 3;7:43772. doi: 10.1038/srep43772 (PMC5335608; doi:10.1038/srep43772)
Supplement: Supplemental Information [file srep43772-s1.pdf]

Supplementary Material

The alr-groEL1 operon in *Mycobacterium tuberculosis*: an interplay of multiple regulatory elements

Aadil H. Bhat, Deepika Pathak and Alka Rao[#]

Correspondence: Rao, A: raoalka@imtech.res.in

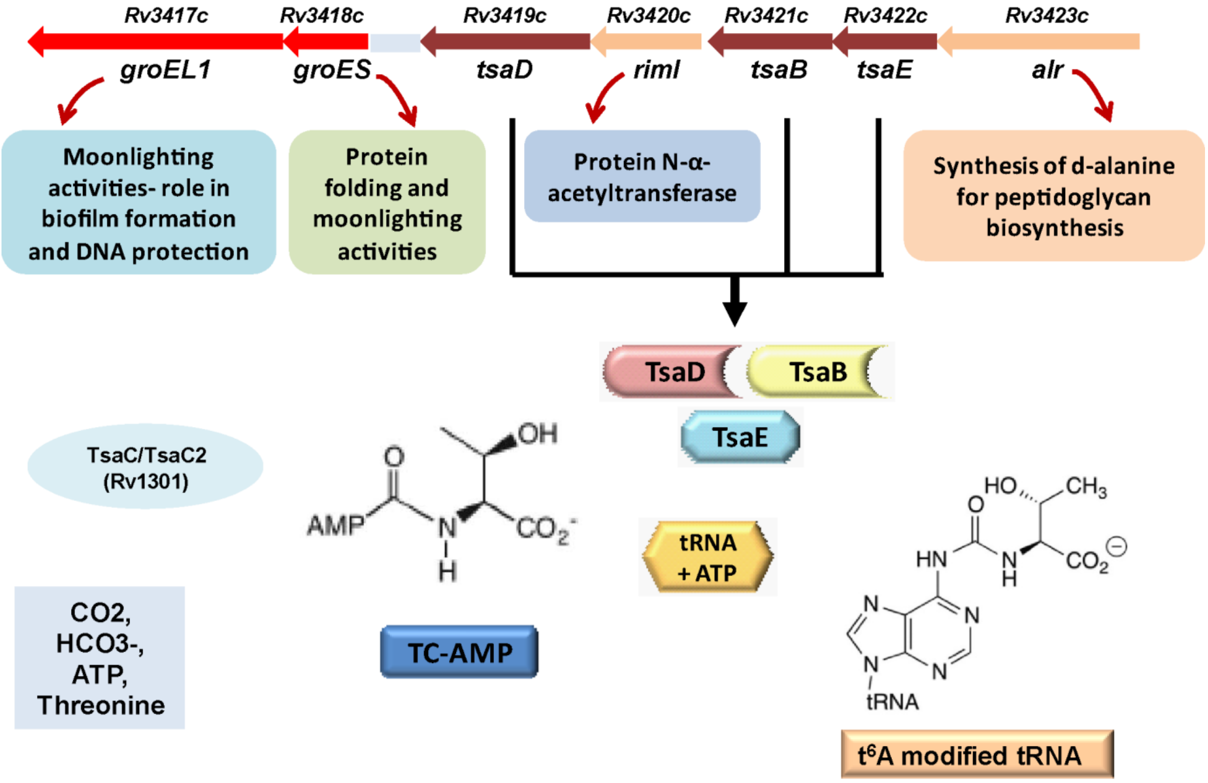

Supplementary Figure S1. Genetic and functional context of homologs of tRNA-A37-t^{6}A biosynthesis machinery in *M. tuberculosis*. Adapted from Thiaville (P. C. Thiaville, D. Iwata-Reuyl, V. de Crécy-Lagard, RNA Biol 11:1529-39, 2014, doi: 10.4161/15476286.2014.992277).

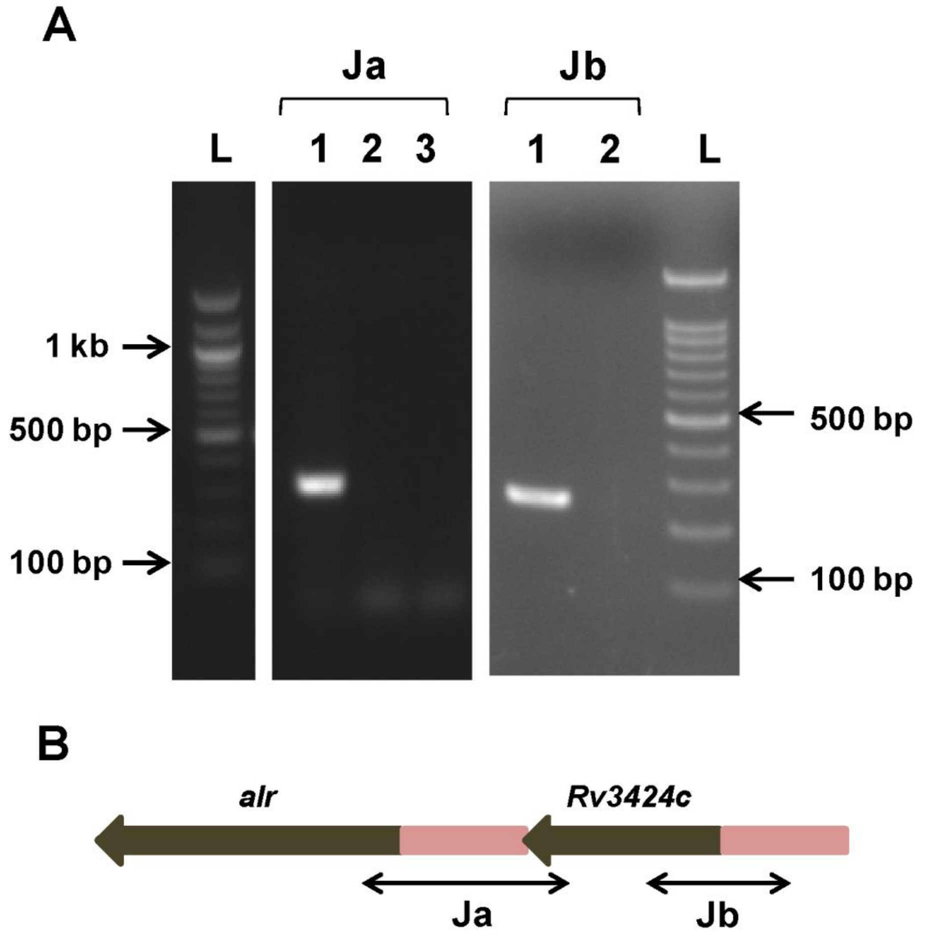

Supplementary Figure S2. RT-PCR of junction between *Rv3423c* (*alr*) and *Rv3424c* using additional primers for unambiguous identification of beginning of the operon (*alr-groEL1*). Panel **A** shows PCR amplified products from genomic DNA template (lane 1 in Ja section), cDNA template (lane 2 in Ja and lane 1 in Jb section) and RT- control (lane 3 in Ja section and lane 2 in Jb section). Combinations of primers used to amplify cDNA at gene-intergene junctions are shown in panel **B** (Supplementary Table S1).

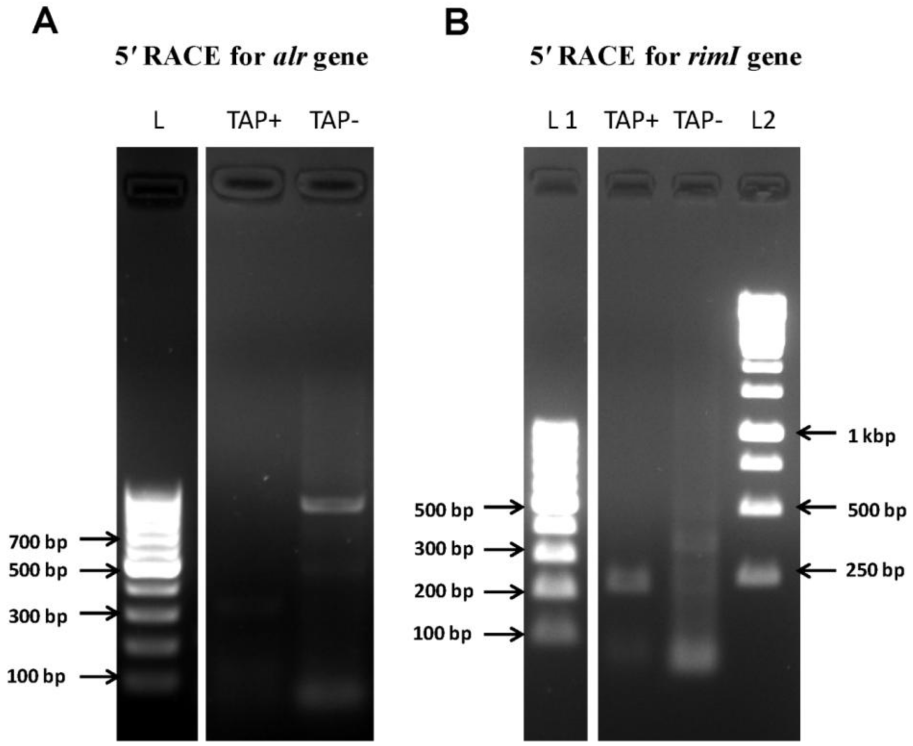

C

TSS ‘G’ at -263 position relative to GTG codon of *alr* gene

gcgttgatggtgcgaagcgaccgccaaagcccatacccgtgcag**TGA**tgcacgggcgcaa
TAGGTTcacttcccccg**G**aactgcggcagtctagggcgaatagtacacactgcaactgtt
tcctgccacgggtgatgggccgcggtcaagctacgctcccgtcaattcatgccggccgat
ccgcactctattgacgccaaccatcatgcggcaccggcaatctgagccaaacaccgggtg
atcggcaaccaaccaccgagccacaggacagtcacgccgacacggctgcagcccagcacc
gcgacccggggcaatcgggcacaggccggaatactgctca**GTG**aaacggttctgggagaa

D

TSS ‘T’ at -275 position relative to ATG codon of *tsaD* gene

atcttcgccggccggatgccaagccgctggcggtttgcac**GTG**acggccgacaccgagcc
cgtcaccatcggcgcgctgacgcgcgcggacgcccagcggtgcgccgagctggaggccca
gctgttcgtcggtgacgatccctggccgccggcggcgttcaaccgcgaactggccagccc
gcacaaccactatgtgggtgcgcgcagcggtggcacgctggtcggt**TACGCT**ggaatc**T**c
gcggttgggccgaacaccgccgttcgagtacgaggtgcacaccatcggcgtggacccggc
ctaccaggggcggggcatcggccgtcggttgctgcgcgaactgctggactttgccagggg
tggtgtggtctacctggaggtccgcaccgataatgacgccgctcttgcgctgtatcgcag
cgtgggattccagcgggtcggcttgcgccggcgatattaccgggtcagcggcgccgacgc
gtacacgatgcgtagggattcgggggacccgtc**ATG**acgacagtcttgggcatcgaaacc

Supplementary Figure S3. Transcription start site mapping using 5′ RLM RACE. RACE was done with *M. tuberculosis* H37Rv total RNA using random hexamers in cDNA synthesis. Two reactions were performed in parallel, one was TAP-treated (TAP+) while the other one (TAP-) was kept as mock. Bands from TAP+ reactions were sequenced. RACE products obtained using 5′ generacer and Rv23R **(A)** or TsaDUR **(B)** reverse primers. Rv23R primer was designed complimentary to the region just downstream of *alr* GTG start codon while TsaDUR primer was designed at the start codon of *tsaD* gene. TSS was identified as G base at -263 position relative to start codon of *alr* **(C)** and as base T at -275 position with respect to the *tsaD* start codon **(D)**. TSSs and the associated -10 hexamer sequences are shown as bold underlined uppercase letters.

Transcription start site (TSS upstream of *alr* gene) mapping attempted with Primer Extension Analysis (PEA)

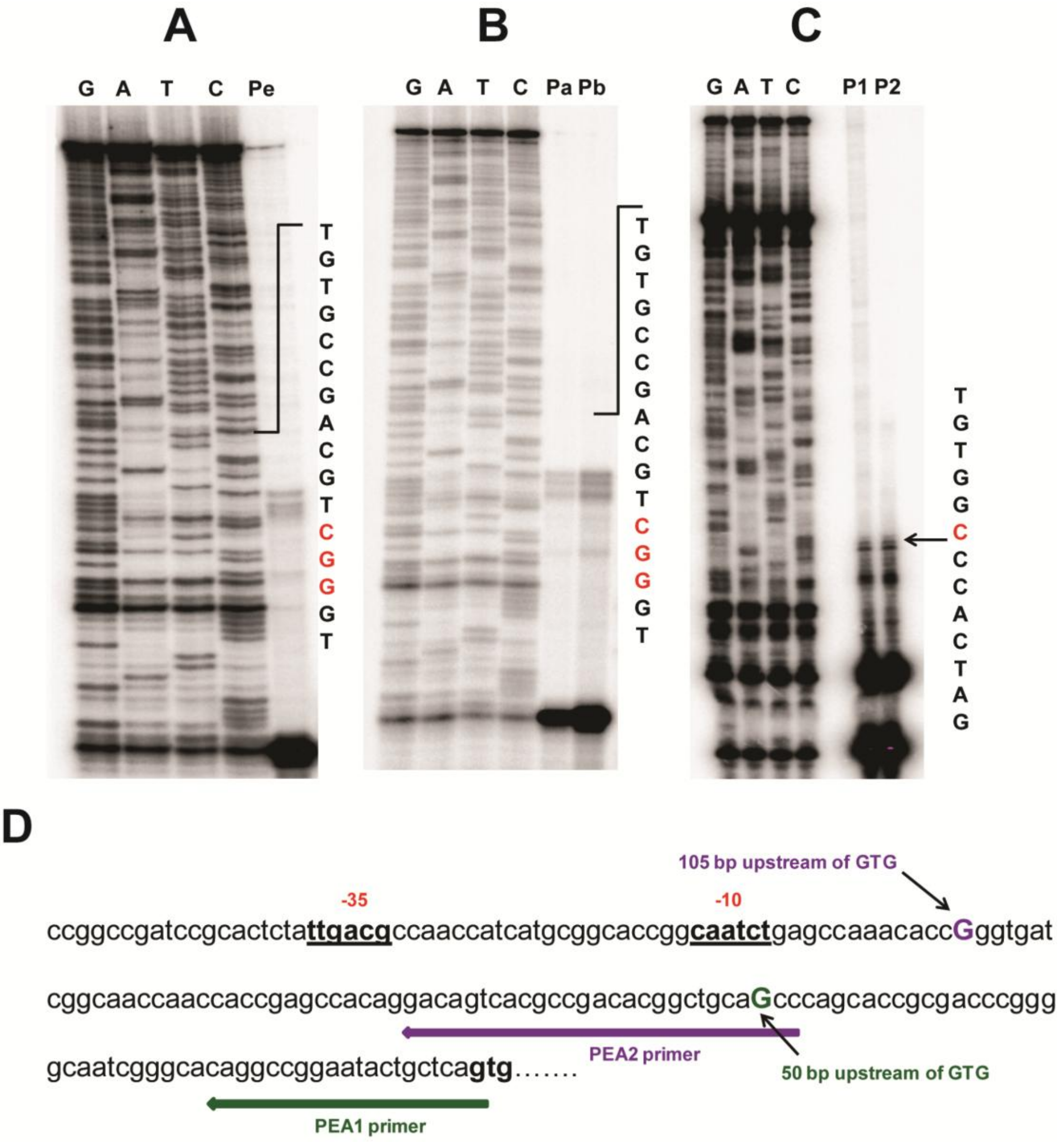

Supplementary Figure S4. Identification of Transcription start site (TSS) using primer extension analysis (PEA) of total RNA isolated from exponential phase *M. tuberculosis* H37Rv. Different primers were designed against the upstream region of the *alr* gene and radiolabeled at 5' ends. PE was performed at different temperatures. **(A)** PE reaction done at 50°C (lane Pe) using PEA1 primer, **(B)** PE reaction carried out at 58°C (lane Pa) and 60°C (lane Pb) using PEA1 primer, **(C)** PE reaction done at 63°C (in duplicate in lanes P1, P2)

using PEA2 primer that overlaps the PEA1 extended product and extends further upstream of PEA1 primer. PEA1 (5'- GTTCATCGATCTGAGCAGTATTCCGGCCTG); PEA2 (5'-GGCTGCAGCCGTGTCGGCGTGACTGTC). Panel **D** shows the position of primer binding in the upstream region of the *alr* gene. See note below for Methodology.

Note: Methodology of Primer Extension Analysis

The radiolabeled oligonucleotides were extended with reverse transcriptase to form a single stranded DNA using the standard protocol [44]. Nearly 5 µg total RNA from *M. tuberculosis* H37Rv was purified and denatured at 70°C for 15 min. Antisense primers were designed against the 5' region of the *alr* gene and end-labeled with [γ-P^{32}] ATP using T4 polynucleotide kinase (Fermentas). A 30-mer PEA1 primer (5'- GTTCATCGATCTGAGCAGTATTCCGGCCTG) was complimentary to nucleotide positions -29 to 1 bp starting from the initiation codon while another 23-mer PEA2 (5'-GCAGCCGTGTCGGCGTGACTGTC) primer was complimentary to -52 to -75 bp nucleotide positions upstream of start codon. Two picomoles of radiolabeled primer were added to the denatured RNA and hybridized for at least 3 h at 55°C. RNA/primer hybrids were resuspended in primer extension buffer subsequent to precipitation with sodium acetate and ethanol. Maxima reverse transcriptase was then added to extend the primers at 58°C or 60°C or 63°C for 1 h. Primer extension product was purified with phenol/chloroform/isoamyl alcohol. The product was analyzed on a 6% denaturing polyacrylamide gel containing 7 M urea, with the help of a labeled sequencing ladder (GATC) made using the same radiolabeled primer.

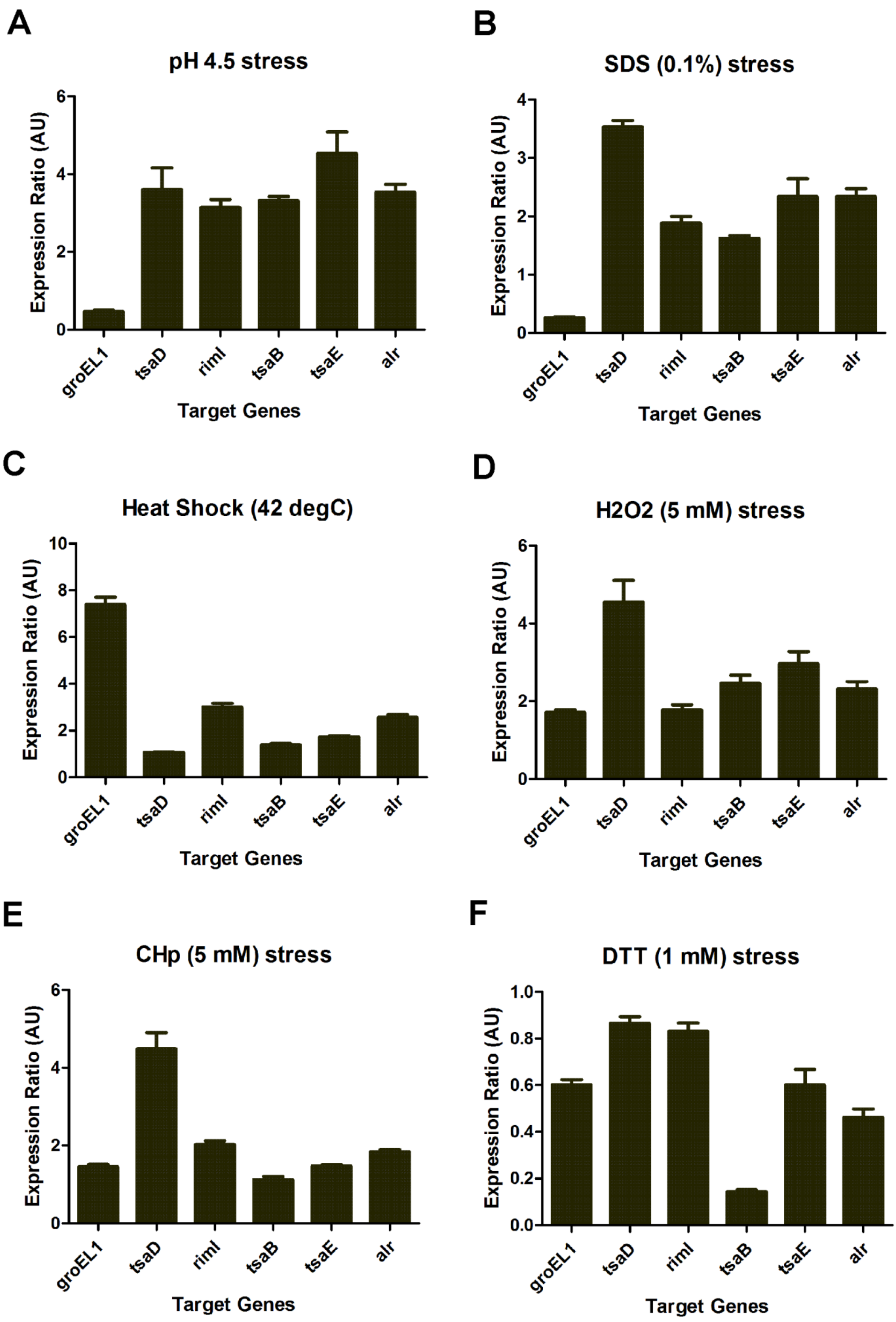
A
pH 4.5 stress
Expression Ratio (AU)
0
2
4
6
groEL1
tsaD
rimI
tsaB
tsaE
alr
Target Genes
B
SDS (0.1%) stress
Expression Ratio (AU)
0
1
2
3
4
groEL1
tsaD
rimI
tsaB
tsaE
alr
Target Genes
C
Heat Shock (42 degC)
Expression Ratio (AU)
0
2
4
6
8
10
groEL1
tsaD
rimI
tsaB
tsaE
alr
Target Genes
D
H2O2 (5 mM) stress
Expression Ratio (AU)
0
2
4
6
groEL1
tsaD
rimI
tsaB
tsaE
alr
Target Genes
E
CHp (5 mM) stress
Expression Ratio (AU)
0
2
4
6
groEL1
tsaD
rimI
tsaB
tsaE
alr
Target Genes
F
DTT (1 mM) stress
Expression Ratio (AU)
0.0
0.2
0.4
0.6
0.8
1.0
groEL1
tsaD
rimI
tsaB
tsaE
alr
Target Genes

Supplementary Figure S5. Relative expression of the constituent genes of the *alr-groEL1* operon under various stress conditions. RT-qPCR was done on total RNA isolated from *M. tuberculosis* H37Rv cultures, subjected to various stresses including heat shock at 42°C, SDS (0.1%), pH 4.5, H_2O_2 (5 mM), cumene hydroperoxide (5 mM), DTT (1 mM). In all cases, the bacteria were kept under stress for approx. 2 h except for heat shock which was given for 30 min. The results (fold change observed) were normalized to the expression of the 16S rRNA reference gene. The results shown are the representative of three independent experiments (with P value ≤ 0.05) done in triplicates. The qPCR results were analyzed using REST 2009 software version 1 (MW. Pfaffl, GW. Horgan, L. Dempfle, Nucleic Acids Res 30, e36, 2002, doi: 10.1093/nar/30.9.e36).

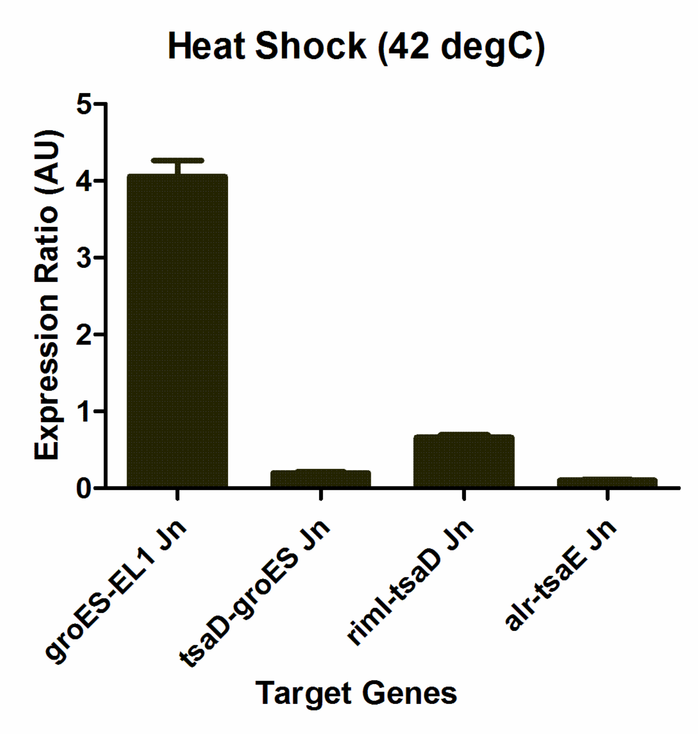

Supplementary Figure S6. Relative expression of different junctions of *alr-groEL1* operon under heat shock at 42°C for 30 min. The bars represent fold change in treated samples relative to control sample. The data shown is the representative of two independent experiments, each performed in triplicate. Mean ± SEM is plotted for each sample.

Supplementary Table S1. Primers used in this study.

No.	Primer	Sequence	Used for
1	JP1F	5’- GCACCGGCAATCTGAGCCAAAC - 3'	
2	JP1R	5’- CGGTGCGGTAATGCCATCAG - 3'	
3	JP2F	5’- CGAAGGCGACGAGGCGATTT - 3'	
4	JP2R	5’- TACCCTTGGCCAGCACCGTCTT - 3'	
5	JP3F	5’ - CGACTGCTGGACCACAACAG - 3'	
6	JP3R	5’ - GTCCAGGTCGGCCATTGTGA - 3'	
7	JP4F	5’- TCTGGTAGCCGCGGTGAACT - 3'	
8	JP4R	5’- CCGAACATGCTCGTCGACAC - 3'	
9	JP5F	5’- CGCGGACGTGTTGACCATGAAG - 3'	Reverse Transcription PCR
10	JP5R	5’- ACCAGGTGATTCGGCATTCG - 3'	
11	JP6F	5’- TGGCAATCGGCTAACCCCTG	
12	JP6R	5’- CGTGCCGACAGGATCAGGTA	
13	JP7F	5’- GGAGGGTGACACCGTCATCT	
14	JP7R	5’- AGTGCCTGCGCCAAGATGGT	
15	JP8F	5’- CTCGGTGGTGGTCAACAAGG	
16	JP8R	5’- CCTACTCCTCAAGGGCACCA	
17	16S F	5’- GTGGCGAACGGGTGAGTAAC - 3'	
18	16S R	5’- CATCAGGCTTGCGCCCATTG - 3'	
19	JaF	5’- AAGTAAGCTTCACTGTCGGCGTTGATG	
20	JaR	5’- GTTCATCGATCTGAGCAGTATTCCGGCCTG	RT-PCR of 5’ end of alr gene and upstream
21	JbF	5’- GTCTGCTGTGCCGGTTTGGTCT	
22	JbR	5’- GATCCCGGTGGTCAACGCTA	
23	AlrU1F	5’- GCAC*TCTAGA*AGCGGCCTCATCCCTATTC	
24	AlrU2F	5’- GCAC*TCTAGA*TAGCCGCGTTGTCCAATGC	
25	AlrU3F	5’- TCAC*TCTAGA*CGCTCCCGTCAATTCATGC	Promoter Cloning
26	AlrU4F	5’- TCAC*TCTAGA*ACAGGACAGTCACGCCGACA	
27	AlrUR	5’- GTTC*AAGCTT*CTGAGCAGTATTCCGGCCTG	

28	TsaEU1F	5’- TCAC*TCTAGA*CGGCGATGACCGTGAAATGTG	
29	TsaEU2F	5’- GCAC*TCTAGA*CCACGCGACACCAATCTGGC	
30	TsaEUR	5’- GTTC*AAGCTT*GATACCCTCACGGCTCAACG	
31	RimIU1F	5’- TCAC*TCTAGA*TTAGCCACTCCGACACCAGGAT	
32	RimIU2F	5’- CTAG*TCTAGA*TCACAATGGCCGACCTGGAC	
33	RimIU3F	5’- TCAC*TCTAGA*GCAAACCATCGGTGACACGC	
34	RimIU4F	5’- CTAC*TCTAGA*GGGATTCGTACGGTCGGACC	
35	RimIU5F	5’- CTAG*TCTAGA*CTGGTAGCCGCGGTGAACTG	
36	RimIUR	5’- GTTC*AAGCTT*ATGGTGACGGGCTCGGTGTC	
37	TsaDU1F	5’- CTAG*TCTAGA*GCTGGCGGTTTGCACGTGAC	
38	TsaDU2F	5’- GCAC*TCTAGA*GCTGGTCGGTTACGCTGGAAT	
39	TsaDU3F	5’- GCAC*TCTAGA*CGATAATGACGCCGCTCTTGC	
40	TsaDUR	5’- GTTC*AAGCTT*GACTGTCGTCATGACGGGTCC	
41	GroESU1F	5’- CTAC*TCTAGA*CCGAGTGTGTGGCGTTGTTG	
42	GroESU2F	5’- GCAC*TCTAGA*CGGACATTGCACCTGGCGTA	
43	GroESUR	5’- GTTC*AAGCTT*TCACCTTCGCCACGATTGGAG	
44	Qt23F	5’- CGGTGACCGTCAAGGTGGATAC	
45	Qt23R	5’- TCGTCAGGCTTGTCGGCGTAA	
46	Qt22F	5’- GACCTGCTGAGTGAGCTGGACT	
47	Qt22R	5’- TCCTGGTGTCGGAGTGGCTAAC	
48	Qt21F	5’- CCGCGTGCAAATAAGCACCGTC	
49	Qt21R	5’- CACGTTGGGAGTCAGCCGTT	
50	Qt20F	5’- ACACCGCCGTTCGAGTACGA	RT-qPCR
51	Qt20R	5’- TCATTATCGGTGCGGACCTCCA	
52	Qt19F	5’- CCGCTATGCGTTCAGCTTCTCC	
53	Qt19R	5’- CCGTACCGCCTTCATGGTCAAC	
54	Qt17F	5’- TGGGCACCGAGTTGGAGTTCA	
55	Qt17R	5’- CGGGAAGCGAGCTGATCTTGTC	
56	Qt24F	5’- TTAGCCGCGTTGTCCAATGCC	
57	Qt24R	5’- CGGTCGCTTCGCACCATCAA	

58	Qt16SF	5'- AGCGGCGGAGCATGTGGATTA	
59	Qt16SR	5'- CCCAACATCTCACGACACGAGC	
60	Rv23_terF	5'- CTAGCATATGGTGACCGTCAAGGTGGATAC	
61	Rv23_terR	5'- TCGTCAGGCTTGTCGGCGTAA	
62	Rv19_terF	5'- TCGACATATGGACATTGCACCTGGCGTAC	Terminator Cloning
63	Rv19_terCF	5'- GGACGAATGCCGAATCACCTGG	
64	Rv19_terR	5'- CCACGATTGGAGCCCTCCAC	
65	TsynA	5'- AGAAAAAAAAAAGCCCCGCGATTGCGGGGCTTTTTTTTT	
66	TtrpA	5'- AGCCCGCCTAATGGCGGGCTTTTTTTT	

Supplementary Table S2. Transcription Start Sites (TSSs) identified in the *alr-groEL1* operon

Name of Gene	TSS	Position on Genome	Position relative to start codon of downstream gene	Method used to identify the TSS	Reference
Rv3418c	G	3837458	170 bp	RNA-seq	Cortes et al.[3] and Shell et al.[2]
Rv3418c	T	3837459	171 bp	Primer Extension	Aravindhan et al.[1]
Rv3418c	C	3837524	236 bp	Primer Extension	Aravindhan et al.[1]
Rv3419c	T	3838864	275 bp	RNA-seq	Cortes et al.[3] and Shell et al.[2]
Rv3419c	T	3838864	275 bp	5′ RLM-RACE	This study
Rv3420c	A	3839615	553 bp	RNA-seq	Shell et al.[2]
Rv3422c	A	3841394	1197 bp	RNA-seq	Cortes et al.[3] and Shell et al.[2]
Rv3422c	G	3841418	1221 bp	RNA-seq	Shell et al.[2]
Rv3423c	A	3841843	423 bp	RNA-seq	Cortes et al.[3]
Rv3423c	G	3841684	263 bp	5′ RLM-RACE	This study

Supplementary Table S3. Detailed description of individual regulatory elements of *alr-groEL1* operon

Promoter elements

S. No	Name of Promoter Fragments	Downstream Gene Regulated	Position on Genome	Size (bp)	[-10 TANNNT consensus]/ (general bacterial -10 box)[a]	Position of -10 box[b]	TSS[c] (bp)	Putative TF[d]	Source/Reference
1	pCVAlrU1	Rv3423c	3841420-2004	584	CAA[TAGGTT]	274 bp	G at -263	Rv1033c	This study
2	pCVAlrU2[g]	Rv3423c	3841420-1886	466	CAA[TAGGTT]	274 bp	G at -263	Rv1033c	This study
3	pCVAlrU3	Rv3423c	3841420-1608	188	-	-	-	Rv1033c	This study
4	pCVAlrU4	Rv3423c	3841420-1499	79	-	-	-	Rv1033c	This study
5	pCVTsaEU1[g]	Rv3422c	3840197-0621	424	TGG(TGAAAT)	367 bp	-	Rv0047c	This study
6	pCVTsaEU2	Rv3422c	3840197-0535	338	-	-	-	Rv0047c	This study
7	pCVRimIU1	Rv3420c	3839062-9787	725	[TATCTT]	35 bp	-	Rv0967	This study
8	pCVRimIU2	Rv3420c	3839062-9545	483	[TATCTT]	35 bp	-	Rv0967	This study
9	pCVRimIU3	Rv3420c	3839062-9397	335	[TATCTT]	35 bp	-	Rv0967	This study
10	pCVRimIU4	Rv3420c	3839062-9324	262	[TATCTT]	35 bp	-	Rv0967	This study
11	pCVRimIU5[g]	Rv3420c	3839062-9182	120	[TATCTT]	35 bp	-	Rv0967	This study
12	pCVGroESU1	Rv3418c	3837288-8214	926	**TATAGA**	179 bp	**T at - 171**	Rv2374c	This study/[1]
13	pCVGroESU2	Rv3418c	3837288-7565	277	**TATAGA**	179 bp	**T at - 171**	Rv2374c	This study/[1]
14	pCVTsaDU1	Rv3419c	3838589-9089	500	*GGT[TACGCT]*	281 bp	*T at - 275*	Rv0967	This study/[2,3]
15	pCVTsaDU2[g]	Rv3419c	3838589-8899	310	*GGT[TACGCT]*	281 bp	*T at - 275*	Rv0967	This study/[2,3]
16	pCVTsaDU3	Rv3419c	3838589-8729	140	-	-	-	Rv0967	This study

I-shaped terminator (hairpin)-like *cis*-regulatory elements

S. No.	Transcription Terminator like Fragment	Upstream Gene Regulated	Position on Genome	Size (bp)	Predicted Terminator Sequence [US (Right leg of Stem)- B (Bulb)- DS (Left Leg of Stem)- T (Tail)][e]	Position (from stop codon present upstream)	Structure Type[e,f]	Other features
1	**TsaDF**	Rv3419c	3837555-7432	123	cgguuccgggg- cacg- ccccgg(acc)gaccg- ccaacuccgg	178 bp	I-shaped	

2	**TtsaDR**	Rv3419c	3837555-7280	275	cgguuccgggg- cacg- ccccgg(acc)gaccg- ccaacuccgg	178 bp	I-shaped	Contains some promoter elements
3	**CTalr**	Rv3423c	3840806-0962	156	-	-	-	Coding sequence of *alr* gene

- Not known/ Not detected

[a] Consensus TANNNT identified by motif discovery using MEME [3]. Consensus motif in bold was identified previously [1].

[b] Position of -10 box with respect to translational start site.

[c] Italicized letters denote TSS annotated by genome wide TSS mapping using Illumina sequencing [3]. TSS in bold letters was determined previously [1].

[d] ChIP sequence data from Minch KJ [4].

[e] Data acquired from: Mitra et al., and Unniraman et al. [5,6].

[f] Palindromic structures with non-canonical U-trails are termed I-shaped.

[g] Minimal promoter fragments identified in our study.

References

1. Aravindhan, V. *et al.* Mycobacterium tuberculosis groE promoter controls the expression of the bicistronic groESL1 operon and shows differential regulation under stress conditions. *FEMS Microbiol. Lett.* **292,** 42–49 (2009).
2. Shell, S. S. *et al.* Leaderless Transcripts and Small Proteins Are Common Features of the Mycobacterial Translational Landscape. *PLoS Genet.* **11,** (2015).
3. Cortes, T. *et al.* Genome-wide mapping of transcriptional start sites defines an extensive leaderless transcriptome in Mycobacterium tuberculosis. *Cell Rep.* **5,** 1121–1131 (2013).
4. Minch, K. J. *et al.* The DNA-binding network of Mycobacterium tuberculosis. *Nat. Commun.* **6,** 1–10 (2015).
5. Mitra, A., Angamuthu, K. & Nagaraja, V. Genome-wide analysis of the intrinsic terminators of transcription across the genus Mycobacterium. *Tuberculosis (Edinb).* **88,** 566–75 (2008).
6. Unniraman, S., Prakash, R. & Nagaraja, V. Conserved economics of transcription termination in eubacteria. *Nucleic Acids Res.* **30,** 675–684 (2002).

Supplementary Table S4. Expression values (Mean ±SEM) used for heatmap generation

	groEL1	***tsaD***	***rimI***	***tsaB***	***tsaE***	***alr***
**pH 4.5 stress**	-2.14 ±0.13	3.597 ±0.571	3.14 ±0.212	3.318 ±0.111	4.536 ±0.549	3.536 ±0.203
**SDS 0.1% stress**	-3.82 ±0.17	3.533 ±0.108	1.883 ±0.116	1.62 ±0.047	2.338 ±0.306	2.338 ±0.138
**Heat Shock 42°C**	7.396 ±0.314	1.072 ±0.014	3.004 ±0.162	1.382 ±0.062	1.726 ±0.056	2.554 ±0.122
**H2O2 5mM stress**	1.716 ±0.066	4.548 ±0.562	1.778 ±0.135	2.462 ±0.211	2.964 ±0.317	2.318 ±0.187
**CuH2O2 5mM stress**	1.456 ±0.058	4.482 ±0.418	2.03 ±0.094	1.124 ±0.082	1.47 ±0.039	1.843 ±0.054
**DTT 1mM stress**	-1.661 ±0.06	-1.156 ±0.039	-1.203 ±0.052	-6.94 ±0.34	-1.67 ±0.18	-2.16 ±0.170
